# Whey Protein Reduces Early Life Weight Gain in Mice Fed a High-Fat Diet

**DOI:** 10.1371/journal.pone.0071439

**Published:** 2013-08-06

**Authors:** Britt Tranberg, Lars I. Hellgren, Jens Lykkesfeldt, Kristen Sejrsen, Aymeric Jeamet, Ida Rune, Merete Ellekilde, Dennis S. Nielsen, Axel Kornerup Hansen

**Affiliations:** 1 Department of Veterinary Disease Biology, Faculty of Health and Medical Sciences, University of Copenhagen, Frederiksberg C, Denmark; 2 Department of Systems Biology, Center for Biological Sequence Analysis, Technical University of Denmark, Lyngby, Denmark; 3 Department of Animal Science, Aarhus University, Denmark; 4 Department of Food Science, Faculty of Science, University of Copenhagen, Frederiksberg C, Denmark; Universidad Pablo de Olavide, Centro Andaluz de Biología del Desarrollo-CSIC, Spain

## Abstract

An increasing number of studies indicate that dairy products, including whey protein, alleviate several disorders of the metabolic syndrome. Here, we investigated the effects of whey protein isolate (whey) in mice fed a high-fat diet hypothesising that the metabolic effects of whey would be associated with changes in the gut microbiota composition. Five-week-old male C57BL/6 mice were fed a high-fat diet ad libitum for 14 weeks with the protein source being either whey or casein. Faeces were collected at week 0, 7, and 13 and the fecal microbiota was analysed by denaturing gradient gel electrophoresis analyses of PCR-derived 16S rRNA gene (V3-region) amplicons. At the end of the study, plasma samples were collected and assayed for glucose, insulin and lipids. Whey significantly reduced body weight gain during the first four weeks of the study compared with casein (P<0.001–0.05). Hereafter weight gain was similar resulting in a 15% lower final body weight in the whey group relative to casein (34.0±1.0 g vs. 40.2±1.3 g, P<0.001). Food intake was unaffected by protein source throughout the study period. Fasting insulin was lower in the whey group (P<0.01) and glucose clearance was improved after an oral glucose challenge (P<0.05). Plasma cholesterol was lowered by whey compared to casein (P<0.001). The composition of the fecal microbiota differed between high- and low-fat groups at 13 weeks (P<0.05) whereas no difference was seen between whey and casein. In conclusion, whey initially reduced weight gain in young C57BL/6 mice fed a high-fat diet compared to casein. Although the effect on weight gain ceased, whey alleviated glucose intolerance, improved insulin sensitivity and reduced plasma cholesterol. These findings could not be explained by changes in food intake or gut microbiota composition. Further studies are needed to clarify the mechanisms behind the metabolic effects of whey.

## Introduction

The prevalence of obesity is increasing dramatically in both Western and developing countries [1]. Therefore nutritional approaches to prevent and control obesity are crucial to fight the epidemic of obesity-related disorders, such as type 2 diabetes and cardiovascular diseases.

Over the last decade, several studies have indicated that dairy consumption has beneficial effects on life style diseases. Elwood and colleagues [2] reported an overall survival advantage from the consumption of milk and dairy foods based on meta-analyses of several studies. Epidemiological studies consistently show that dairy-product intake is inversely related to obesity, as determined by body mass index [3]–[5], and the metabolic syndrome [6]–[8] which is defined as a cluster of risk factors for cardiovascular disease and type 2 diabetes [9], [10]. Conclusions from intervention studies are, however, rather inconsistent, as some studies demonstrate a beneficial effect of dairy product intake on the amount of body fat [11], [12] whereas other studies show no effect on body weight and other parameters related to the metabolic syndrome [13], [14]. Various milk components have been suggested to be beneficial, including the milk proteins whey and casein. Both are high quality proteins and contain all the essential amino acids [15]. Recent studies indicate that whey protein in particular reduces body weight and improves insulin sensitivity in rodent models [16]–[19] as well as in humans [20]–[22].

Within the last years, it has become increasingly evident that the gut microbiota (GM) plays an important role in conditions such as obesity and type 2 diabetes [23]–[25]. Diet influences the composition of the GM; especially high-fat diet [26], [27] and non-digestible carbohydrates [28], named prebiotics, are known to have a strong impact on GM. Only a few studies have been published regarding the influence of dietary protein quality or quantity on the GM. Yet, whey protein has been shown to increase the quantity of fecal lactobacilli and bifidobacteria when compared with casein in a study by Sprong *et al.* [29]. Thus, whey protein potentially influences metabolic health, and a possible mechanism may be through changes in the GM. In this study, we therefore investigated the metabolic effects of whey protein compared with casein in mice fed a high-fat diet, and we hypothesised that the effects of whey protein on the various hallmarks of the metabolic syndrome would be associated with changes in the GM composition.

## Results

### Body weight, feed efficiency, and organ weights

**Figure 1:**
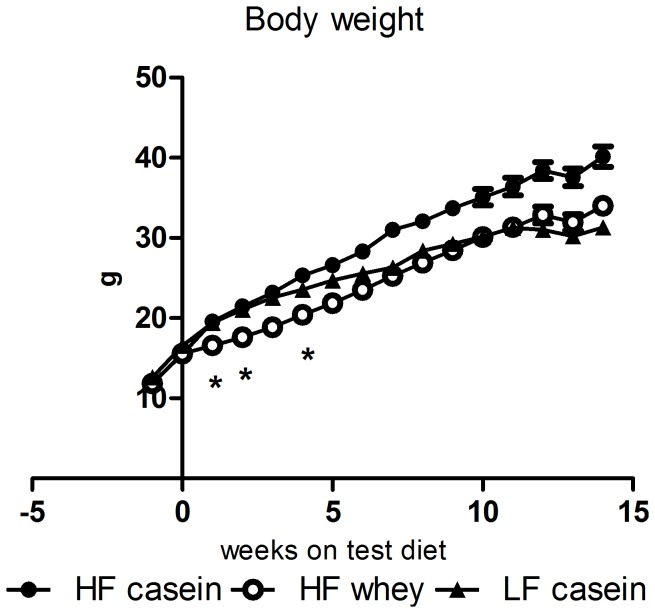
Body weight during 14 weeks of dietary intervention. The body weight of HF whey was lower than HF casein at all times after week 0 (P<0.05). The weekly weight gain was lower in HF whey than HF casein at week 1, 2, and 4 (*, P<0.05). Data is presented as mean ±SEM and compared by one-way ANOVA. HF casein, high-fat diet with casein (n = 15), HF whey, high-fat diet with whey protein isolate (n = 15), LF casein, low-fat diet with casein (n = 10).

Replacement of casein with whey in the diet markedly decreased weight gain during the early weeks of the study. After the first week of diet intervention, mice on high-fat (HF) whey had gained 1.0±0.3 g of body weight compared to 4.1±0.3 g in the HF casein group and 2.9±0.3 g in the low fat control (LF casein) (HF whey vs. HF casein: P<0.001; HF whey vs. LF casein: P<0.001). The difference in weekly weight gain declined the following three weeks and thereafter the weight gain was similar between the two HF groups. At the end of the study, this resulted in a significantly lower final body weight in the HF whey group (34.0±1.0gram) compared to the HF casein group (40.2±1.3gram) (P<0.001) (Figure 1 and Table 1).

**Table 1:** Food intake and feed efficiency.

	HF casein	HF whey	LF casein
Weight gain (g/cage) 0–4 weeks	49.1±1.5^a^	24.3±3.9^b^	35.3±2.1^a,b^
Energy intake (kJ/cage) 0–4 weeks	6723.3±81.0^a,b^	7479.1±344.1^a^	5860.3±283.5^b^
Feed efficiency (mg/kJ) 0–4 weeks	7.31±0.24^a^	3.24±0.44^b^	6.01±0.06^a^
Weight gain (g/cage) 4–14 weeks	74.1±7.0^a^	68.0±0.2^a^	38.5±2.1^b^
Energy intake (kJ/cage) 4–14 weeks	15060.1±685.3	15381.8±1035.0	13681.0±47.2
Feed efficiency (mg/kJ) 4–14 weeks	4.90±0.26^a^	4.46±0.30^a^	2.81±0.14^b^

Results not sharing a common superscript differ significantly (P<0.05).

While cumulated energy intake did not differ significantly between HF whey and HF casein group, feed efficiency was reduced in the HF whey group compared to HF casein during the first four weeks (P<0.001) but not during the last 10 weeks (Table 1). Food intake was not at any time point significantly lower in HF whey than in HF casein, indicating that palatability was not compromised when replacing casein with whey (data not shown).

Whey reduced the relative inguinal (subcutaneous) fat pad weight compared to casein (P<0.05, HF casein vs. HF whey) while the relative weights of the epididymal and perirenal/retroperitoneal fat pad (intra-abdominal fat) were unaffected by protein source (Table 2). Consequently, body fat percentage, as estimated by the total amount of measured fat divided by body weight, was not significantly different between HF casein and HF whey (data not shown). Absolute muscle weights (Table 2) and liver weights (data not shown) did not differ between groups.

**Table 2:** Body composition after 14 weeks of dietary intervention.

	HF casein	HF whey	LF casein
Inguinal fat (mg/g)	23.86±1.85^a^	18.38±1.64^b^	13.62±1.10^b^
Epididymal (mg/g)	30.45±1.36^a^	30.61±2.33^a^	18.58±1.12^b^
Perirenal and retroperitoneal fat (mg/g)	10.09±0.30^a^	9.52±0.76^a^	6.47±0.44^b^
Gastrocnemius and soleus muscles (mg)	154±5	156±5	159±5
Quadriceps muscles (mg)	180±8	182±8	191±12

Results not sharing a common superscript differ significantly (P<0.05).

### Plasma lipids

Fasting plasma total cholesterol was significantly lowered by whey (P<0.001, HF casein vs. HF whey) to a concentration not different from the LF casein group whereas fasting plasma triacylglycerols and non-esterified fatty acids were similar in all groups (Table 3).

**Table 3:** Plasma lipid profile and glycemic parameters.

	HF casein	HF whey	LF casein
Cholesterol (mM)	4.49±0.14^a^	3.66±0.15^b^	3.44±0.15^b^
Triacylglycerols (mM)	0.92±0.05	0.96±0.05	0.95±0.04
Non-esterified fatty acids (mM)	1.65±0.10	1.73±0.10	1.80±0.10
Glucose (mM)	16.88±0.69^a^	14.75±0.68^a,b^	12.89±0.68^b^
HbA1c (%)	4.31±0.04	4.39±0.04	4.27±0.06
Insulin (pM)	762.1±149.6^a^	318.4±33.1^b^	221.0±29.6^b^
QUICKY	0.23±0.005^a^	0.25±0.004^b^	0.26±0.004^b^

Results not sharing a common superscript differ significantly (P<0.05).

HbA1c, glycated haemoglobin. QUICKI, Quantitative Insulin Sensitivity Check Index.

### Glycemic parameters

**Figure 2:**
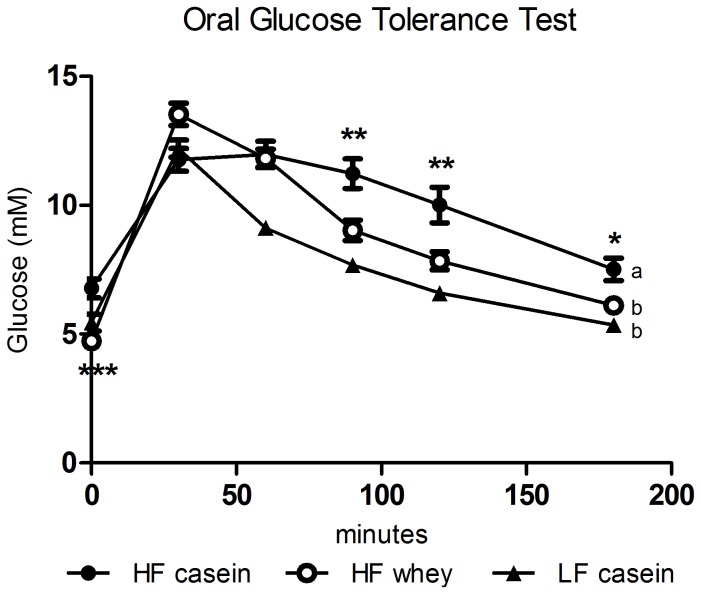
Oral glucose tolerance test after 12 weeks of dietary intervention. Glucose clearance was significantly improved by whey (P<0.05, HF casein compared to HF whey, Repeated Measurements ANOVA and Tukey's Post Hoc test). Asterisks indicate time points were blood glucose in HF whey is significantly lower than in HF casein (one-way ANOVA). * P<0.05, ** P<0.01, *** P<0.001 in comparison to HF casein. Data is presented as mean ±SEM. HF casein, high-fat diet with casein (n = 15), HF whey, high-fat diet with whey protein isolate (n = 15), LF casein, low-fat diet with casein (n = 10).

Fasting blood glucose prior to glucose administration during the OGTT was significantly lower in the HF whey group compared to HF casein (P<0.001). Even though blood glucose rose to a significantly higher level at time  = 30 minutes (P<0.05), blood glucose was significant lower in the HF whey group compared to the HF casein group at time  = 90 (P<0.01), 120 (P<0.01), and 180 (P<0.05) minutes after glucose administration (Figure 2). While AUC calculations did not reveal any significant difference between HF casein and HF whey due to the higher peak of HF whey at time  = 30 (data not shown), ANOVA Repeated Measurements revealed a significant difference in blood glucose curves between HF casein and HF whey (P<0.05) (Figure 2). At the end of the study, fasting insulin was significantly reduced by whey feeding (P<0.01) and fasting plasma glucose was also reduced although it failed to reach statistical significance (P<0.10, HF casein compared to HF whey) (Table 3). Consequently, insulin sensitivity in the HF whey group, as estimated by the Quantitative Insulin Sensitivity Check Index (QUICKI), was markedly improved compared with HF casein (P<0.01) (Table 3). HbA1c was not significantly different between any of the three groups (Table 3).

### Fecal microbiota

**Figure 3:**
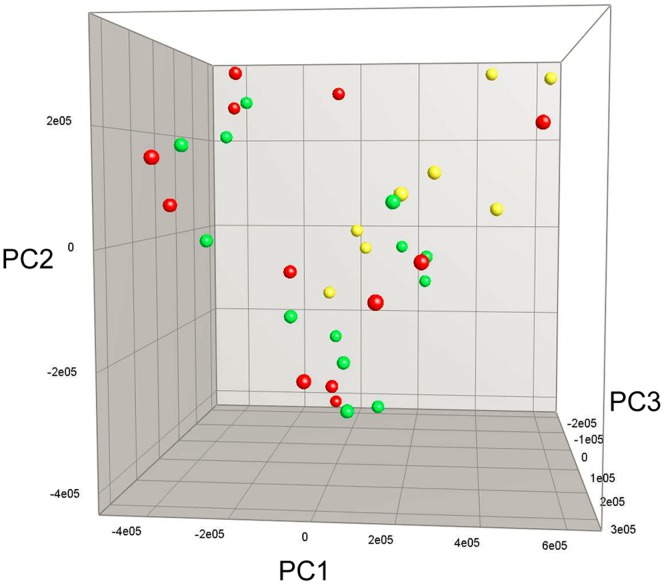
Fecal microbiota profile after 13 weeks of dietary intervention. Principal Component Analysis Plot based on Denaturing Gradient Gel Electrophoresis profiles of 16S rRNA gene PCR-derived amplicons of fecal samples. Each ball represents the relative bacterial composition of one fecal sample. No difference in gut microbiota composition was detected between HF casein (red balls) and HF whey (green balls) while the composition significantly differed between high-fat and low-fat groups (LF casein, yellow balls) (P<0.05). HF casein, high-fat diet with casein, HF whey, high-fat diet with whey protein isolate, LF casein, low-fat diet with casein.

The composition of the GM, analyzed by denaturing gradient gel electrophoresis (DGGE), was similar between all groups at the beginning of the experiment and after 7 weeks of diet intervention (data not shown). After 13 weeks there was a significant difference between high- and low-fat diets (P<0.05) while protein source did not affect GM composition (Figure 3).

## Discussion

In this study, we showed that whey reduces body weight gain, alleviates glucose intolerance, improves insulin sensitivity and reduces plasma cholesterol in mice fed a high-fat diet. Interestingly, whey had an acute but not chronic effect on weight gain. After the first week the effect gradually ceased but the weight difference persisted throughout the study period. During the initial weeks, even the control group on a low-fat diet gained more weight than the mice fed a high-fat diet with whey. While many studies have shown a reductive effect of whey on final body weight the time course of the effect is typically not discussed. As estimated by the published graphs of body weight gain in related studies, timing is a factor that should be taken into consideration when exploring the mechanisms of whey-reduced body weight gain. As an example, female whey-fed C57BL/6J mice did not gain weight during the first 10 days of the high-fat diet intervention while the high-fat control mice immediately gained weight [18]. In another mouse study, whey reduced weight gain gradually during the diet intervention [30]. In one rat study, the most profound reduction in body weight gain by whey was seen during the first week of the diet intervention when whey protein was compared to red meat protein [19] while Royle et al. [17] reported reduced weight gain by whey compared to casein during both early and later weeks of the intervention. Thus, published data indicate that whey protein feeding requires a couple of weeks of adaption before normal body weight gain is re-established.

Accumulated food intake was neither reduced by whey in our, nor in the above-mentioned studies. Our data does not suggest that changes of the composition of the GM are responsible for the reduced body weight gain either as no difference was found on the DGGE between casein and whey fed mice. However, early GM changes cannot be ruled out completely as faeces was sampled after seven and thirteen weeks of intervention where body weight gain was unaffected by protein source. The scarcity of studies on the effect of dietary protein on faecal GM composition might be explained by an efficient absorption of protein in the small intestine causing possible changes in the proximal gut to be non-detectable when the intestinal content reaches the rectum.

As both whey and casein are high-quality protein sources it seems unlikely that the reduced weight gain of whey-fed mice is due to suboptimal nutrition. However, the absorption kinetics of whey and casein are rather different. Whey proteins enter the small intestine as intact proteins and are relatively quickly absorbed. Casein forms a clot in the acid environment of the stomach which causes a slower absorption [31]. One explanation of the acute effect of whey on weight gain could be that the postprandial amino acid delivery to the liver is too rapid to be fully utilised by the body, at least until a certain adaptation has occurred. This explanation is supported by the results of a study by Lacroix et al. [32] where healthy volunteers received a meal with different [^15^N]-labelled milk protein fractions. During the following eight hours plasma amino acids, proteins, urea and urinary urea were measured. Milk soluble protein isolate (whey protein) caused a high but transient hyperaminoacidemia which concomitantly lead to an earlier and higher transfer of dietary nitrogen to urea when compared to casein and total milk protein. Total postprandial deamination was increased by whey protein indicating a higher degree of “protein-waste” and therefore a suboptimal ability to support (muscle) growth. These findings in humans are based on a single meal whereas the mice in our study were fed ad libitum. Mice and rats do however tend to eat in a meal-like pattern with three larger meals eaten during the dark phase [33], [34]. Catabolism of amino acids is an energy-consuming process [35], [36], hence an increased influx of amino acids into the urea cycle as a consequence of hepatic amino acid overload after whey intake could therefore explain the decreased feed efficiency at week 0–4 seen in the present study. After a couple of weeks, a physiological adaptation seems to occur and the amino acids of whey are apparently more successfully utilized for growth in consistency with the similar weight gain during the last period of the study and the similar final muscle mass to HF casein. In our study the mice were rather young (five weeks old) and displaying a high growth rate when placed on the test diets which might have made them even more susceptible to a possible decreased utilisation of whey protein. In the study by Lacroix et al. [32] total milk protein ingestion resulted in the best dietary nitrogen utilisation suggesting that the natural combination of casein and whey in milk is the most beneficial for growth (80∶20 ratio in cow's milk).

Irrespective of the similar weight gain during the last ten weeks of the study and the same relative amount of intra-abdominal fat, whey significantly alleviated other parameters of the metabolic syndrome compared to casein at the end of the study. Glucose intolerance and insulin resistance were attenuated in agreement with the findings in female C57BL/6 mice by Shertzer *et al.* [18]. Fasting plasma cholesterol was also lowered by whey consistent to the results of a human dietary intervention study [21]. These beneficial effects of whey could not be explained by changes in feed efficiency or GM composition in our study. Whey differs, however, from casein in other ways than absorption kinetics. While casein is a well-defined pure protein, whey consists of a heterogeneous group of soluble proteins that exhibits some variation depending on the manufacturing process. For instance, glycomacropeptide, a whey protein which has been shown to improve several metabolic parameters as investigated by Royle et al. [17], is removed by ion exchange and therefore is not found in the diet tested in the present study. Generally, whey is rich in branched-chain amino acids including leucine which have been shown to decrease obesity, improve glycemic control and decrease plasma cholesterol in high-fat fed C57BL/6J mice [37]. Casein is rich in phosphor which might influence mineral absorption when caseino-phosphopeptides are formed during casein digestion. Since calcium binds to the caseino-phosphopeptides [38] there might be less free calcium available for synthesis of intestinal fat-calcium salts, leading to a smaller fat excretion and thus higher apparent fat absorption, in casein-fed mice compared to whey-fed mice as suggested by Pilvi et al. [30]. Thus, the higher level of branch-chained amino acid and the potentially decreased fat absorption might add to the explanation of the metabolic effects of whey compared to casein observed in this study. As our experiment was designed to study the effects in a high-fat fed model and therefore did not include a low-fat whey-group we cannot rule out a potential interaction between whey and dietary fat content.

In conclusion, whey significantly alleviated several conditions associated with the metabolic syndrome in mice fed a high-fat diet. The mechanisms behind the early reducing effect of whey on body weight as well as the other metabolic effects remain to be studied further.

## Materials and Methods

### Ethics Statement

The study design was approved by the National Committee for Animal Experimentation, Ministry of Justice, Copenhagen, Denmark (License Number: 2007/561-1434 C1).

### Experimental design

40 male C57BL/6NTac mice (Taconic, Laven, Denmark) were purchased at the age of three weeks and housed in groups of five with free access to food and water. Temperature was maintained at 20–24°C, the relative humidity was 55%±10% and the light was automatically switched off from 6 pm to 6 am. During two weeks of acclimatization, all mice were fed an Altromin 1324 standard rodent diet (Brogaarden, Denmark). At the age of five weeks, the cages were matched by body weight and divided into three experimental groups (“HF casein”, “HF whey”, and “LF casein”) to be fed a high-fat diet with casein, a high-fat diet with whey protein isolate, and a low-fat control diet with casein, respectively, as described below. The groups were fed the respective test diets for 14 weeks. Body weights were recorded weekly and food intake was recorded twice weekly.

### Test diets

The HF casein group and the LF casein group were fed the widely used casein-based D12492 and D12450B with 60%, respectively 10%, of energy from fat (Research Diets Inc., New Brunswick, NJ, USA). The HF whey group received a high-fat diet similar to HF casein except for the protein source which was replaced with whey protein isolate (custom made based upon D12492 by Research Diets) (Table 4). The whey protein isolate consisted of undenatured soluble whey proteins processed by ion exchange and ultrafiltration (NZMP, New Zealand). Amino acid profiles of both protein sources are listed in Table S1.

**Table 4:** Detailed diet composition^a^ of the three experimental groups.

Group and product code	HF casein (D12492)	HF whey (D10082504)	LF casein (D12450B)
Casein, 30 Mesh (g)	200	0	200
Whey protein isolate^b^ (g)	0	189	0
L-Cystine (g)	3	3	3
Corn Starch (g)	0	0	315
Maltodextrin 10 (g)	125	125	35
Sucrose (g)	68.8	68.8	350
Cellulose, BW 200 (g)	50	50	50
Soybean Oil (g)	25	25	25
Lard (g)	245	245	20
Mineral Mix S10026 (g)	10	10	10
DiCalcium Phosphate (g)	13	13	13
Calcium Carbonate (g)	5.5	5.5	5.5
Potassium Citrate, 1H_2_O (g)	16.5	16.5	16.5
Vitamin Mix V10001 (g)	10	10	10
Choline Bitartrate (g)	2	2	2
Energy (kJ/g)	21.4	21.4	15.5
Protein (kJ%)	18	18	18
Carbohydrate (kJ%)	20	20	71
Fat (kJ%)	62	62	10

a Diets were formulated and produced by Research Diets Inc. (New Brunswick, NJ, USA).

b Alacen 895, NZMP, New Zealand.

### Fecal sampling and analyses

At week 0, 7, and 13 weeks fecal pellets were collected in autoclaved micro tubes (Brand, Wertheim, Germany) immediately after defecation. If the mouse did not defecate spontaneously when restrained it was kept in a clean cage until defecation. The samples were initially kept on ice and subsequently stored at −80°C until further analyses. DNA extraction, Polymerase Chain Reaction (PCR), denaturing gradient gel electrophoresis (DGGE), and data analyses were performed as previously described [39]. In brief, bacterial DNA was extracted using the QIAamp DNA Stool Mini Kit (Qiagen, Hilden, Germany). Quality and concentration of the extracted DNA was verified on a NanoDrop 1000 Spectrophotometer (Thermo Scientific, USA). Genetic material was then amplified by PCR, using primers specific to the V3 region of the 16S rRNA gene and genetic material was separated by means of DGGE on a polyacrylamid gel containing a 30%–65% denaturing gradient (100% corresponds to 7 M urea and 40% formamide). The DGGE is further explained in Figure S1.

### Oral glucose tolerance test and HbA1c

An oral glucose tolerance test (OGTT) was performed after 12 weeks of diet intervention. After an overnight fast (10 hours) the mice were gavaged orally with a glucose solution of 2 mg glucose/g body weight, and blood glucose was measured before dosing and after 30, 60, 90, 120, and 180 minutes using Freestyle Mini Glucometer (Hermedico, Copenhagen, Denmark). At week 13, HbA1c was measured on a Siemens DCA Vantage Analyzer (Siemens Healthcare Diagnostics, Ballerup, Denmark) by transferring 1 µl full blood from a tail vein puncture to the supplied collection cassette.

### Blood sampling and tissue collection

After 14 weeks of diet intervention, the mice were anesthetised with a Hypnorm/Dormicum mixture (Hypnorm: 0.315 mg/ml Fentanyl, 10 mg/ml Fluanisone; VetaPharma Ltd, Leeds, UK. Dormicum: 5 mg/ml Midazolam; Roche A/S, Hvidovre, Denmark) injected subcutaneously as a 1∶1∶2 sterile water solution (0.006 ml/g body weight) after an overnight fast (10 hours). Blood was collected from the periorbital plexus in EDTA coated micro tubes (Milian, Geneva, Switzerland) and immediately centrifuged for plasma. The mice were killed by cervical dislocation. Liver, fatty and muscular tissues were weighed and kept on dry ice until storage at −80°C or fixated in formalin for future studies.

### Plasma analyses

Plasma glucose, cholesterol, non-esterified fatty acids and triacylglycerols were analysed on an automatic analyser (Cobas Mira Plus, Roche Diagnostics, Hvidovre, Denmark) using reagents from Horiba ABX (Montpellier Cedex 4, France). Plasma insulin was measured by a commercial mouse ELISA kit (Mercodia, Uppsala, Sweden). The QUICKI index was calculated based on the logarithmic transformation: 1/(log insulin [µU/ml]+log glucose [mg/dl]).

### Statistics

Results are presented as mean ±SEM unless otherwise stated. The Prism software package (Version 5.02, GraphPad Software Inc., San Diego, CA, USA) and SAS Analyst (Version 9.2, SAS Institute Inc., Cary, NC, USA) were used to manage and statistically analyse the data. Experimental groups were compared by one-way ANOVA and Tukey's Post Hoc test. For the OGTT, both one-way ANOVA on area under the curve (AUC) calculations and Repeated Measurements ANOVA with Tukey's Post Hoc test were applied. When relevant, data (glucose, insulin) was log transformed to normality. DGGE profiles were analysed for clustering by Dice similarity coefficient with a band position tolerance and optimization of 1% using the Unweighted Pair Group Method with Arithmetic averages clustering algorithm (UPGMA) and Principal Component Analysis (PCA) (BioNumerics, Version 4.5, Applied Maths, Sint-Martens-Latem, Belgium) as previously described [39]. The significance level was set at 0.05.

## Supporting Information

Figure S1 **Cluster analysis similarity tree of fecal microbiota.** Dendrogram of DGGE profiles representing 16S rRNA gene-derived amplicons of fecal samples collected from C57BL/6 mice fed high-fat diet with casein (red circles), high-fat diet with whey (green circles) and low-fat diet with casein (yellow circles), respectively, for 13 weeks. Each horizontal lane represents one fecal sample (indicated by an arrow) and each band (black line) on a lane represents in principle one bacterial species. The larger the distance between two samples in the dendogram the more different their compositions are. The mice clustered strongly after gel which was included as a factor in the statistical analysis. Principal Component Analysis revealed a significantly different clustering between high- and low fat groups (P<0.05) demonstrating different fecal microbial composition while there was no effect of protein source. DGGE, denaturing gradient gel electrophoresis.
(PDF)

Table S1 **Amino acid profile of casein and whey protein.**
(DOCX)
