# Supplementary figures and images for: Whey Protein Reduces Early Life Weight Gain in Mice Fed a High-Fat Diet
Source: PLoS One. 2013 Aug 6;8(8):e71439. doi: 10.1371/journal.pone.0071439 (PMC3735523; doi:10.1371/journal.pone.0071439)

Figure S1. Cluster analysis similarity tree of fecal microbiota.

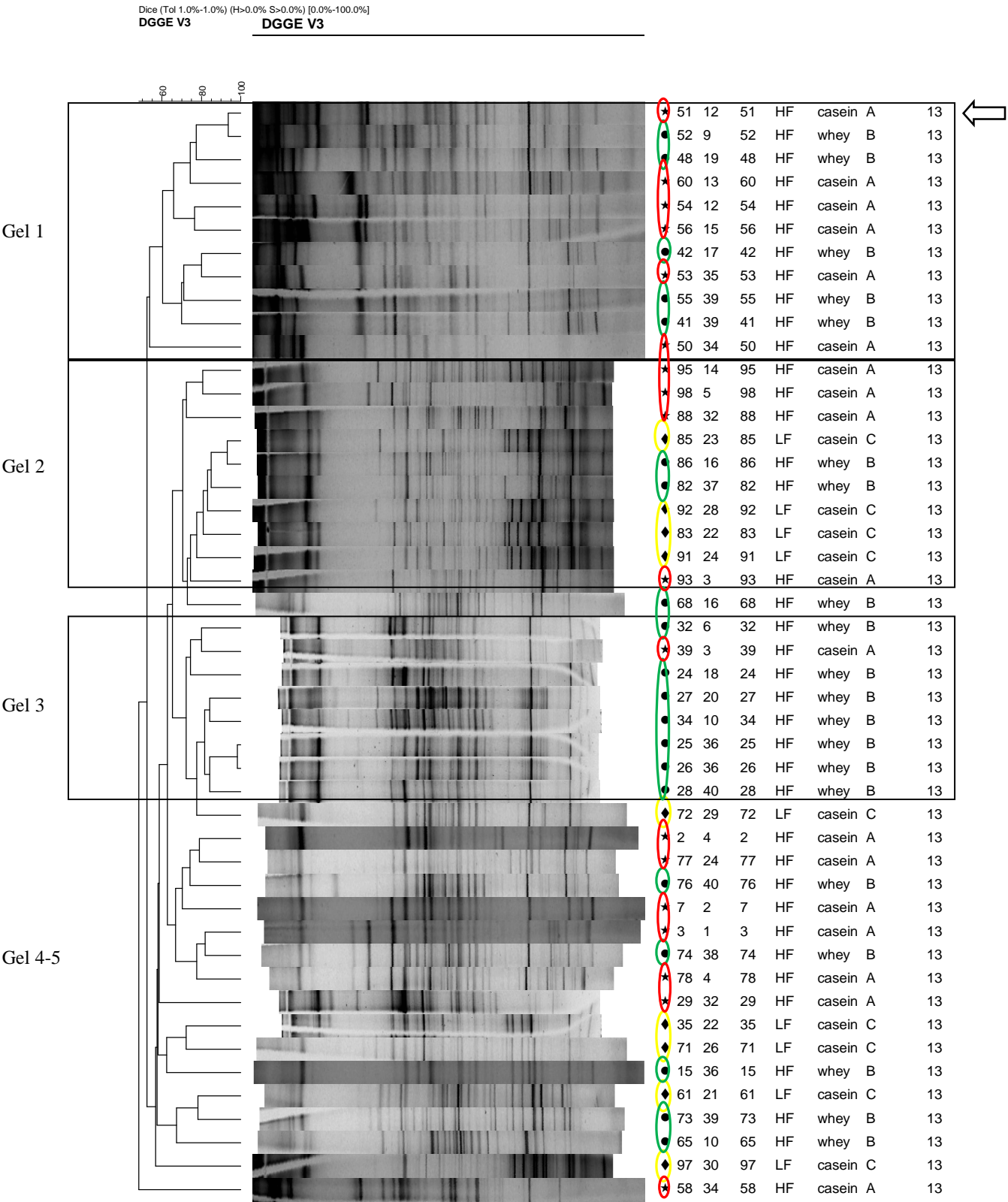

Supplement: Figure S1 — Cluster analysis similarity tree of fecal microbiota. Dendrogram of DGGE profiles representing 16S rRNA gene-derived amplicons of fecal samples collected from C57BL/6 mice fed high-fat diet with casein (red circles), high-fat diet with whey (green circles) and low-fat diet with casein (yellow circles), respectively, for 13 weeks. Each horizontal lane represents one fecal sample (indicated by an arrow) and each band (black line) on a lane represents in principle one bacterial species. The larger the distance between two samples in the dendogram the more different their compositions are. The mice clustered strongly after gel which was included as a factor in the statistical analysis. Principal Component Analysis revealed a significantly different clustering between high- and low fat groups (P<0.05) demonstrating different fecal microbial composition while there was no effect of protein source. DGGE, denaturing gradient gel electrophoresis. (PDF) [file pone.0071439.s001.pdf]
