# Supplementary material for: Whey Protein Reduces Early Life Weight Gain in Mice Fed a High-Fat Diet
Source: PLoS One. 2013 Aug 6;8(8):e71439. doi: 10.1371/journal.pone.0071439 (PMC3735523; doi:10.1371/journal.pone.0071439)
Supplement: Table S1 — Amino acid profile of casein and whey protein. (DOCX) [file pone.0071439.s002.docx]

Table S1. Amino acid profile of casein and whey protein.

| Protein source | Casein^a^ | Whey^b^ |
| --- | --- | --- |
| Isoleucine (g/100g protein) | 4.3 | 6.3 |
| Leucine (g/100g protein) | 9.0 | 14.3 |
| Lysine (g/100g protein) | 7.5 | 11.2 |
| Methionine (g/100g protein) | 2.9 | 2.4 |
| Phenylalanine (g/100g protein) | 4.8 | 3.8 |
| Threonine (g/100g protein) | 4.1 | 5.3 |
| Tryptophan (g/100g protein) | 1.2 | 2.4 |
| Valine (g/100g protein) | 5.3 | 5.6 |
| Histidine (g/100g protein) | 2.6 | 2.0 |
| Alanine (g/100g protein) | 2.9 | 5.7 |
| Arginine (g/100g protein) | 3.4 | 3.0 |
| Aspartic acid (g/100g protein) | 6.9 | 12.5 |
| Cysteine (g/100g protein) | 0.7 | 4.0 |
| Glutamic acid (g/100g protein) | 21.7 | 17.6 |
| Glycine (g/100g protein) | 1.7 | 1.8 |
| Proline (g/100g protein) | 10.1 | 4.5 |
| Serine (g/100g protein) | 5.7 | 4.5 |
| Tyrosine (g/100g protein) | 5.2 | 4.2 |

^a^Lactic Casein Alacid 720, 30 Mesh, NZMP, New Zealand

^b^Alacen 895 Whey Protein Isolate, NZMP, New Zealand
